# New products

**DOI:** 10.1155/S1463924694000167

**Published:** 1994


Journal of Automatic Chemistry, Vol. 16, No. 4 (July–August 1994), pp. 143–149

### New products

### Hydride generation atomic absorption

GBC Scientific Equipment has released another report in its AA Applications series. The report describes the advantages of electical heating of the quartz cell over flame heating of the cell for hydride work. Electrical heating provides more accurate temperature control, as well as improved sensitivities. Comparisons are made for a number of common hydride forming elements and mercury.

*Copies from Orry Dugdale, GBC Scientific Equipment UK Ltd, 13 Frederick Sanger Road, The Surrey Research Park, Guildford, Surrey GU2 5YD, UK. Tel.: 0483 304988; fax: 0483 303071.*

### Process TOC analyser

The model 2100 Process Total Organic Carbon Analyser has been added to the range of TOC measuring instruments available from Sartec. This is a compact, lightweight design with a rugged enclosure suitable for most environments. It is intended for industrial water applications in the ranges 0 to 2 ppm and 0 to 5000 ppm total organic carbon and offers reliability, low maintenance and real-time analysis of process streams.

Fast, continuous response and simple operation make it suitable for a diverse selection of applications. These include duties in the chemical and petrochemical industry, in which the 2100 is used for monitoring wastewater, effluent, condensate, cooling and process water.

Operation is by continuous ultra-violet promoted persulphate oxidation, which coverts organic carbon to carbon dioxide which is measured by an accurate non-dispersive infra-red detector. Output is calibrated to read direct in ppm carbon.

The Model 2100, in its standard NEMA-3R enclosure, measures 600 mm high by 380 mm wide by 210 mm in depth. In this form it is for general purpose use in non-hazardous locations. As an option an air purged model can be supplied for use in Class 1, Division 2 hazardous locations. As a further option an inert purge gas can be used. Options available include sample stream multiplexing, sample handling/filtration systems and sample dilution. Power supply required is 220 Vac 50/60 Hz single phase. Power consumption is 350 watts.

*A detailed leaflet is available from Sartec, Bourne Enterprise Centre, Borough Green, Kent TN15 8DG, UK. Tel.: 0732 884815; fax: 0732 885541.*

### CryoSTM

New from Oxford Instruments is the first commercially available, fully integrated, low temperature system for the STM (Scanning Tunnel Microscope). The system, which is also available in an ultra-low temperature version, was developed by Oxford Instruments in collaboration with WA Technology. Both versions are controlled through the transputer-controlled TOPSystem II system, which, through its open architecture, allows the configuration of a wide range of experiments. Applications include studies of phase transitions, superconductors, adsorbates, molecular manipulation and heavy fermion spectroscopy.

The UHV CryoSTM system is a radical development of the Oxford Instruments Ultrastat cryostat system. It provides an ultra-high vacuum helium-cooled finger upon which a special low-temperature STM is integrated. The ultra-low temperature version is a fully integrated dilution refrigerator system and allows experimentation in the 50 mK temperature region.

The system allows atomic resolution over a wide area and its variable temperature facility allows the study of phase transitions and many other phenomena. The system's in-vacuum tip and specimen exchange provides for easy operation without having to break the vacuum. It includes internal vibration isolation and has a compact design which integrates easily with UHV chambers.

*For further information contact Kevin Hill, Oxford Instruments, Old Station Way, Eynsham, Witney, Oxon OX8 1TL, UK. Tel.: 0865 882855; fax: 0865 881567.*

### P S Analytical appoints European sales manager

With a consistent record for sales growth during recent years, PSA is pleased to announce an organizational change which creates a special responsibility for Europe. Chris Glazier, until recently with Varian, will take over the European territory with its many established distributors, to consolidate sales of existing products and to plan the launch of new products.

PSA offers a range of products to determine elements which are contaminants in aqueous and gaseous media. These include mercury, arsenic, selenium, antimony, all with detection levels in the region of a few ppt. Current global legislation increasingly places a requirement on low levels of detection of these and similar elements, as well as a need to speciate the various elements.

*For any communication regarding the above please contact Paul Stockwell at P S Analytical Ltd, Arthur House, B4 Chaucer Business Park, Kemsing, Sevenoaks, Kent TN15 6QK, UK. Tel.:0732 763416; fax: 0732 761340.*

### Screening or determining trace metals?

Researchers can use TraceLab 50, which combines voltammetric and polarographic measuring techniques,

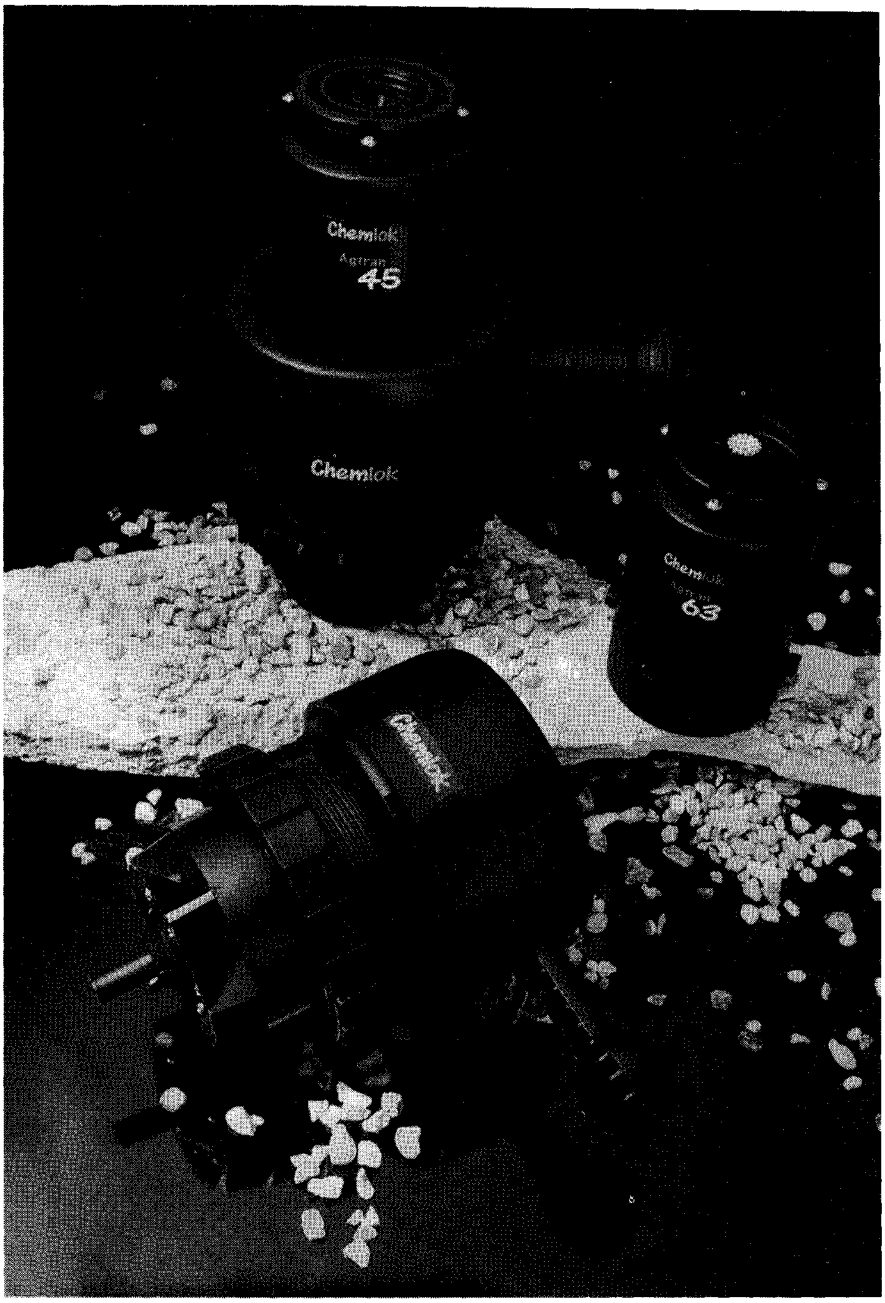

*Growing pressure for safer handling methods with hazardous substances has prompted Chemlok plc, the designers and suppliers of closed fluid handling equipment, to launch Agtran and Biotran, the first of their range of Closed Chemical Transfer Systems. These systems have been designed specifically for use in the agrochemical and water treatment markets.*

*Chemlok consists of a tank and bottle unit which lock together to provide the safe closed transfer of liquids from the donor container into a receiving vessel. A safe, dry break is a feature of the system when the two parts are decoupled. Part loads are no problem either, and an integral rinse device ensures that both the containers and the locking system are thoroughly clean after use. Operators will find it easy and fast to operate and the construction from tough materials will enable Chemlok to withstand rugged use. More information from Chemlok plc, 43 Wolsey Road, East Molesey, Surrey KT8 9EW, UK. Tel.: 081 979 6617; fax: 081 979 0662.*

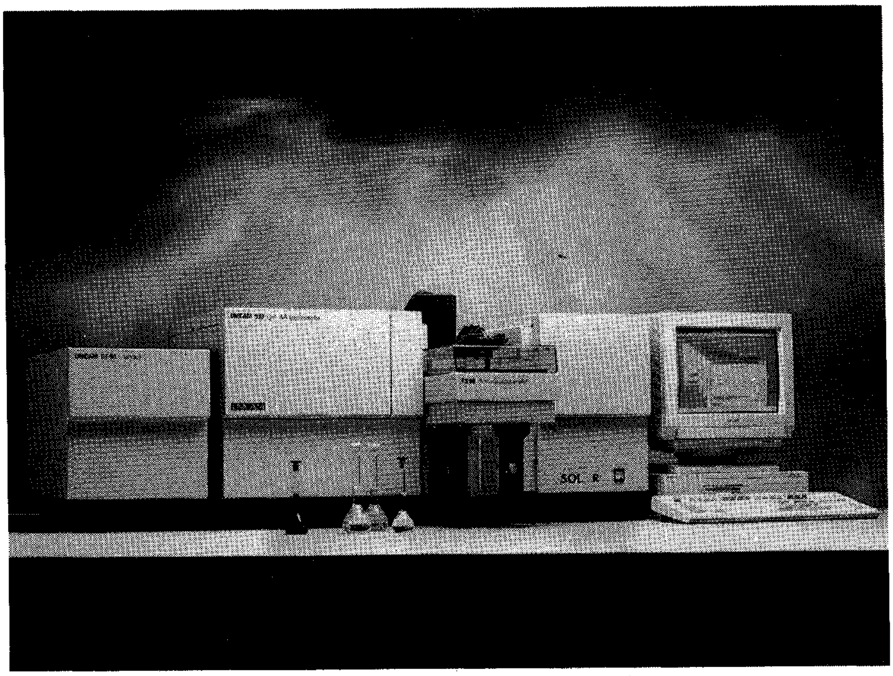

*ATI Unicam's SOLAAR 939QZ. This graphite furnace atomic absorption spectrometer provides AC Zeeman and QuadLine deuterium correction together in one instrument. Details from Paul Carter, ATI Unicam, York Street, Cambridge CB1 2PX, UK. Tel.: 0223 358866; fax: 0223 312764.*

to determine nearly all heavy metals and many organic electroactive compounds. As well as a Mercury Drop Electrode Kit, TraceLab 50 also functions with a rotating disk electrode or solid state electrode. Stripping and direct potential scan modes enable environmental laboratories to rapidly screen a wide range of concentrations.

Set-up only requires an inert gas supply and operation is easy using the Windows-based TraceMaster 5 software. A multitasking facility greatly enhances data processing and enables post-run calculations to be made while samples are being measured. TraceLab 50 has also been designed to ensure maximum safety when working with mercury. Comprising a microprocessor-controlled POL 150 polarographic analyser and a dedicated MDE 150 polarographic stand, TraceLab 50 also occupies minimal benchspace.

*For more information contact Ed Lemon, Radiometer Ltd, The Manor, Manor Royal, Crawley, West Sussex RH10 2PY, UK. Tel.: 0293 517599; fax: 0293 531597.*

### Radiometers

Multi-Sense is a family of radiometers developed by Ultra-Violet Products Ltd; the range includes economical, entry-level units, advanced handheld computer units, desktop systems, dosimeters, UV monitors and scanning monochromators. Among the first products to be launched are two optical radiometers—Multi-Sense 100 and Multi-Sense 200—each designed to be used with a wide range of sensors. Multi-Sense 100 is a low-cost device ideally suited to simple measurement and uses the SEN 100 series sensors. Sensors are available to measure 254, 310, 365, 405, 555 nm, magnetic fields from 0 to 19·99 Tesla and temperature from −50°C to +199·9°C. Multi-Sense 100 is a hand-held unit and there are no external switches. All results are clearly displayed on an LCD which also indicates when the batteries are low.

The Multi-Sense 200 is a more sophisticated optical radiometer which has been designed around a Psion Organiser II handheld computer. By plugging the Multi-Sense module into the Psion and connecting an appropriate sensor, both intensity and dosage measurements can be made. In addition, the Psion has the ability to store results for later review or downloading to a PC. The Multi-Sense 200 will accept the range of SEN 100 sensors with the exception of the magnetic field and temperature sensors.

An additional range of sensors, the SEN 200 series are fitted with interference filters and are precision devices more suited to demanding research and production duties. Wavelengths for these plug-in sensors are 254, 295, 310, 365, 405, 436, 546, 555, 577, 691 and 1014 nm.

*For further information contact Paul Ellwood, Ultra Violet Products Ltd, Science Park, Milton Road, Cambridge CB4 4FH, UK. Tel.: 0223 420022; fax: 0223 420561.*

### Zymark Corporation launches consultation service

Zymark's new Consultation Service Division will respond to the increasing need among laboratories for assistance in developing strategies and guidelines enabling them to reap the benefits of automation as quickly as possible. The service offers assistance in validating hardware, software, the interaction between components, as well as the

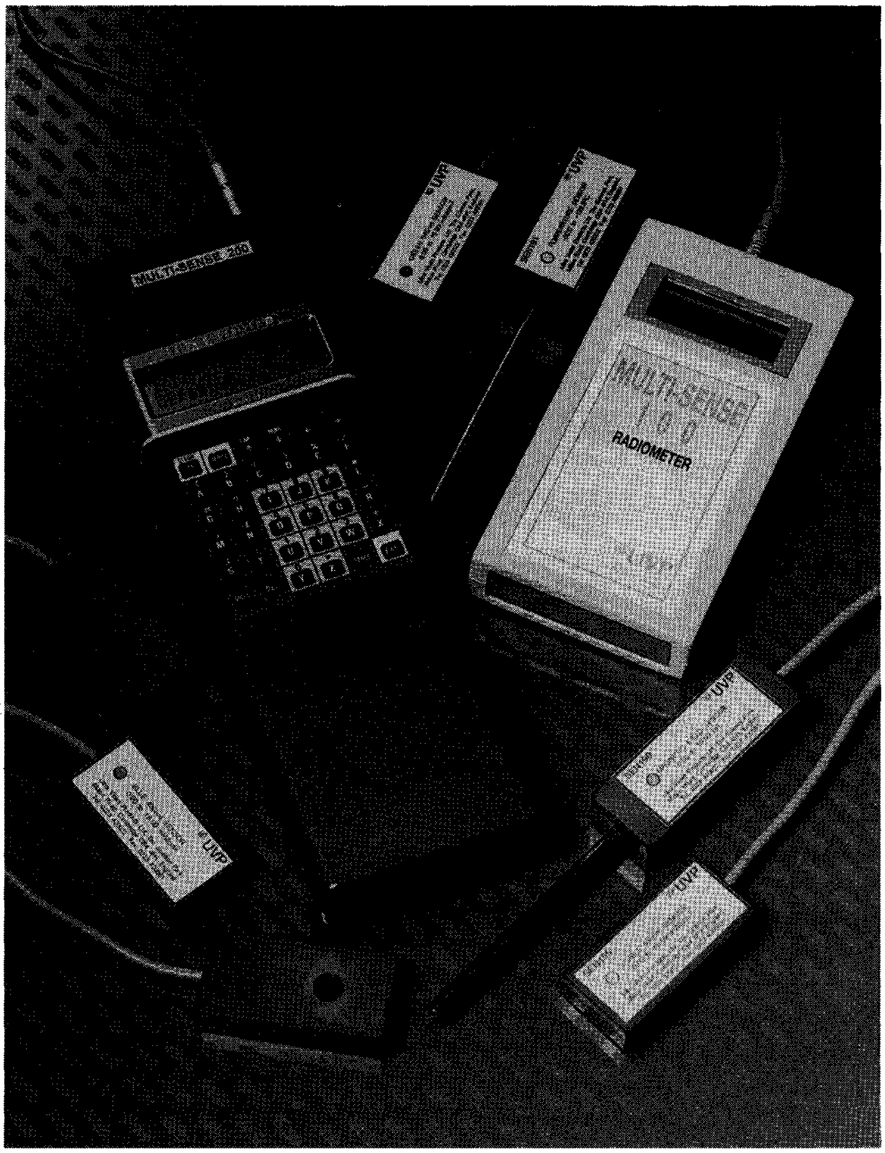

*Multi-Sense—range of radiometers from UVP offering a unique range of options and benefits. Multi-Sense 100 and Multi-Sense 200 are both designed to be used with plug-in sensors* (*see previous page*).

system's overall performance. Validation services are provided for sample preparation systems: Installation Qualification (IQ), Operational Qualification (OQ), and Performance Qualification (PQ); Analytical Method Validation; and System Suitability.

One of the first steps in implementing an automated laboratory system is conducting a comprehensive analysis that assures the scientific integrity for both equipment and procedure. Validation not only ensures the accuracy of the process, but confirms the procedures comply with internal corporate guidelines and Good Manufacturing Practice (GMP) requirements.

Zymark Corporation has designed and installed 2200 robotic systems and thousands of workstation-based automation products for use in the research and analytical laboratory environment.

*Details from Zymark Consulting, Zymark Center, Hopkinton, MA 01748, USA. Tel.: 508 435 9500; fax: 508 435 3439.*

### Swingwirl

Endress & Hauser's new range of flanged flow meters offers cost-effective metering for all standard process measurements. The Swingwirl will measure gas, liquids and steam across a temperature range of −200°C to +400°C at pressures of up to 250 bar. The device offers an accuracy of better than ±0·75%. The loop powered DMV 6336 version uses only one sensor and one board for the complete 15–300 mm size range. The device can be commissioned and range on any duty without the need for special tools, and its traceable calibration remains constant throughout its lifetime, even if the sensor or electronics are exchanged.

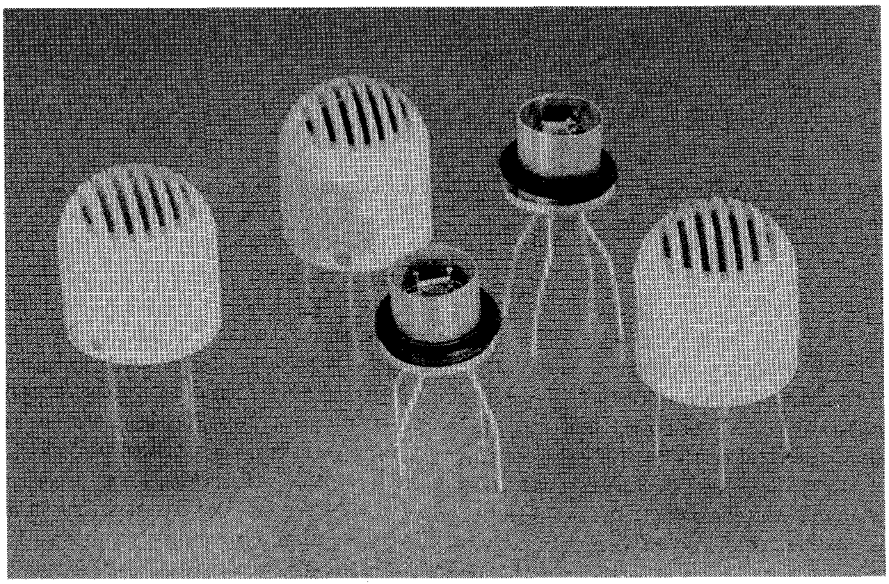

*Typical target gases that can be identified with 'Capteur' sensors are ethylene, methane, propane, butane and general hydrocarbons. Operation is up to 10 000 ppm and in the LEL and occupational health concentration ranges, but sensors could be developed for other specific gases and sensitivities (see below).*

The Swingwirl range offers a choice of body materials, including stainless steel, hastelloy, titanium and monel. Special versions include a high pressure weld-on device, plus 'dual sense', which provides two independent measurements in one pipe assembly.

*Further information from Endress + Hauser Ltd, Ledson Road Manchester M23 9PH, UK. Tel.: 061 998 0321; fax: 061 998 1841.*

### Sensors for low gas concentration

The Capteur range of sensors will detect toxic, flammable and refrigerant gases, including oxygen. The devices are small, light weight and designed to given long service life without the need for routine replacement. They do not contain catalysts or non-consumables which limit conventional sensor life, and are designed for continuous operation within a temperature range of −80° to 250°C.

These Capteur solid state sensors employ second generation oxide semi-conductors, incorporating the latest ceramic fabrication processes combined with thick film printing. Units can be supplied with a heater driven to provide the output independent of ambient temperature changes or flow rates and to a lesser extent the external humidity. No poisonous additives or reagents are incorporated and power consumption is in the range of 500–950 mW, depending upon the target gas.

Three models can be supplied, including a slimline version suitable for high volume applications. Two four-pin versions are also offered, one designed for location in housings approved for pellistors.

*Further information from Sensortechnics UK, 30 Regent Place, Rugby, Warwiskshire CV21 2PN, UK. Tel.: 0788 560426; fax: 0788 561228.*

### A/D interface board and general-purpose Chemstation Software

Hewlett-Packard has added an analogoue-to-digital (A/D) interface board to its range of high performance A/D converters for chromatographic data acquisition. The HP 35900D board is designed to be installed into IBM or IBM-compatible personal computers using industry standard architecture (ISA).

HP has also announced HP 3365 general-purpose ChemStation software to take advantage of the HP 35900D A/D board.

The HP 35900D offers chromatographers two independent channels of ultra-high resolution (24-bit) signal conversion in an integrated design. The product also features eight lines of digital input/output (I/O), which can be used to connect relay devices controlling external timed events such as valve switching, or connect to automatic samplers to input bottle numbers.

The HP 35900D A/D interface board also features: sampling rates from 0·1 to 100 Hz; 200 times the resolving power of most 16-bit boards; a design that eliminates 'cross-talk' and keeps signals isolated and chromatographic separations reproducible; and easy installation as no external grounding or shielding is necessary.

The HP 3365 general-purpose ChemStation software is a PC-based package that, combined with the HP 35900D A/D board, is designed to provide data acquisition and analysis for any chromatographic instrument. The ChemStation is based upon open, industry-standard PC hardware and software architecture and the Microsoft Windows-based software features an easy-to-use graphical user interface and configuration program.

The software's multi-method sequences allow the entire chromatographic process to be automated. The system can be further customized by automating pre- and post-run programs. To speed statistical analysis and custom reporting, information from chromatographic runs can be sent to other software packages.The new software can be purchased separately or bundled with an HP Vectra PC.

*Enquiries to Verena Haller, Hewlett-Packard SA, 150 Route du Nant-d'Avril, CH-1217 Meyrin (GE) 2, Switzerland.*

### PR/SR balances

Mettler Toledo's PR/SR precision balances meet GLP requirements, as well as those of other modern QA systems are met without compromise. All essential features and capabilities are available, particularly in the areas of the control of inspection, measuring and test equipment and documentation. All accumulated data can be reliably acquired, evaluated, documented and used again.

Operation is simple through an alphanumeric terminal and permanently built-in, rapidly recallable weighing applications mean that the PR/SR balances are convenient. The relationship between the weighing speed and the reproducibility can be adapted to match the application. There is a choice between 11 PR models for maximum capacities from 210 to 8100 g with a readability between 0·001 and 1 g. The maximum capacity of the 7 SR balances lies between 8100 and 32 100 g with a readability of 0·1 or 1 g.

*More information from Mettler-Toledo AG, CH 8606 Greifensee, Switzerland.*

### Technical calculation

MathSoft, Inc. has launched Mathcad 5.0 and Mathcad PLUS 5.0, the first products in MathSoft's new, multi-tiered product family. Mathcad 5.0 offers new features and increased usability for mainstream users, while Mathcad PLUS 5.0 is a new edition that delivers both usability and a wider array of maths functions for advanced technical professionals. An alternative to spreadsheets, calculators and programming languages, Mathcad technical calculation software performs numeric and symbolic calculations. Mathcad works like an electronic whiteboard on which equations, text, and graphics can be entered for quick calculation. Users can change equations, values and parameters and watch Mathcad recalculate the answer with automatic unit assignment and conversion. This information can then be printed as it appears on screen with text, graphics and equations in maths notation. Mathcad also serves as a platform for MathSoft's growing line of Electronic Books, which provide access to standard formulas, data and commonly used equations.

Mathcad 5.0 and PLUS 5.0 are available for £195 and £395, respectively. Both editions will run as 32-bit applications under Windows 3.1 and Windows NT

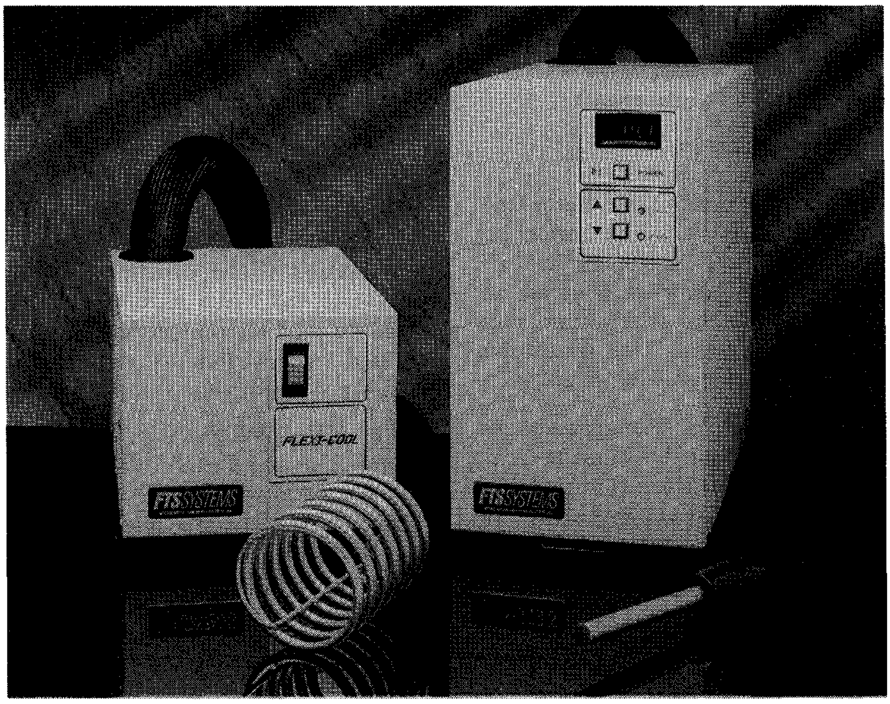

*The FC55 immersion cooler from FTS Systems generates temperatures down to −55°C using non-CFC refrigerant gas in a reliable mechanical refrigeration system. The FC55 can be used in place of dry ice or liquid nitrogen as a cooling source for liquid baths or as a cold trapping surface to trap solvents or other condensates from vapour streams. The FC55 generates cooling power of more than 230 BTUs/hour at −40°C and has a footprint of 10·5 in wide × 16·5 in deep. Details from Thermal Conditioning Division, FTS Systems, Inc., PO Box 158, Rt 209, Stone Ridge, NY 12484, USA. Fax: 914 687 7481.*

compatible systems. They require a 386-based or higher personal computer running DOS 4.0 or later. Mathcad 5.0 requires 4 MB or RAM, 8 MB of free disk to be used as a swap space, and 14 MB of free disk space. Mathcad PLUS 5.0 requires 8 MB of RAM, 8 MB swap space and 16 MB of free disk space. A maths coprocessor is recommended but not required. A 32-bit C or C++ compiler is needed to use the C, C++ programming interface. Mathcad supports the leading C or C++ compilers from companies such as Microsoft, Borland, Watcom and Symantec compilers. MathSoft provides free, unlimited technical support for both products.

*For more information contact Paul Sloane, Vice President of International Sales, MathSoft Europe, Kingswick House, Kingswick Drive, Sunninghill, Berkshire SL4 7BH, UK. Tel.: 0344 23491; fax: 0344 873461.*

### Sensor-array photometers

Dr Bruno Lange (UK) of Camberley, Surrey has announced the introduction of the LASA 10 and LASA 20 sensor-array photometers. The new instruments automatically make all the necessary wavelengths available. With the simultaneous detection of several wavelengths, direct measurement of turbid and coloured samples is possible. Electronic parameters, such as pH, temperature, conductivity and oxygen, do not create any problems. Features of LASA 10 and LASA 20 include:

Reference beam technology.
Precision adjustment.
Automatic zero balancing.
Updated programme module.
Measured value memory.
Mains and stand-alone operation.
Compact size.
RS-232 interface.

LASA 10 has been specifically designed for analysis of the relevant nutrient parameters—COD, total nitrogen, ammonium, nitrite, phosphate and oxygen. LASA 20 also analyses the nutrient parameters, but in addition, analyses heavy metals, various anions such as cyanide, chloride and sulphate, and a range of other industrial parameters. The main application areas are in the sewage treatment, food, brewing, metal, pharmaceutical and paper industries.

*For more information contact Dr Bruno Lange (UK) Ltd, PO Box 93, Camberley, Surrey GU15 1DU, UK. Tel.: 0276 677233; fax: 0276 677307.*

### Science education at Pittcon

Continuing education was the reason that several hundred scientists met for the first Pittsburgh Conference and Exposition on Analytical Chemistry and Applied Spectroscopy in Pittsburgh, Pennsylvania, in 1950. The 45th conference opened on 27 February 1994, at McCormick Place, Chicago. This was the second time the conference has been held here; the first was in 1991. In intervening years it is held in New Orleans and Atlanta.

Some things—besides the location—have changed over the years; instead of a few hundred attendees, Pittcon '94 attracted 30 000 scientists; an exposition of the latest laboratory instrumentation and services has grown from a handful of companies to more than 1000 firms demonstrating instruments and technologies that did not even exist when the first conference was held.

Because of this, the conference long ago outgrew its namesake city.

Some things about Pittcon remain the same as in 1950: the conference is still organized and staffed by volunteers who are members of the Society for Analytical Chemists of Pittsburgh (SACP), and the Spectroscopy Society of Pittsburgh (SSP). Education also remains the focal point of this annual event. Through the SACP and SSP, all profits from the conference are distributed among numerous projects. These include grants and scholarships for students from kindergarten through high school and college; awards to high school teachers for outstanding performance; starter grants to young college professors; technical subscription endowments to libraries; subscriptions to American Chemical Society journals and magazines for student use at universities and colleges; the International Chemistry Olympiad for high school students; awards to outstanding chemists and spectroscopists; and distinctive philanthropic projects on a national level.

The 45th anniversary of the Pittsburgh Conference was marked by a special exhibit of 'instrumental antiquities', prototype or early models of instruments which have proven significant to the growth of analytical chemistry. The exhibiting companies included many which have participated in the conference since its founding, and whose efforts contribute to the ongoing growth in the field.

*Details from the Pittsburgh Conference, Suite 332, 300 Penn Center Blvd, Pittsburgh, PA 15235-5503, USA.*

